# Combined Layer/Particle Approaches in Surface Molecular Imprinting of Proteins: Signal Enhancement and Competition

**DOI:** 10.3390/s18010180

**Published:** 2018-01-10

**Authors:** Nam Van Ho Phan, Hermann F. Sussitz, Eva Ladenhauf, Dietmar Pum, Peter A. Lieberzeit

**Affiliations:** 1 University of Vienna, Faculty for Chemistry, Department of Physical Chemistry, Waehringer Strasse 42, 1090 Vienna, Austria; honamd99@yahoo.com (N.V.H.P.); hermann.franz.sussitz@univie.ac.at (H.F.S.); 2 Institute of Biophysics, Department of Nanobiotechnology, University of Natural Resources and Life Sciences, Vienna, Muthgasse 11, A-1190 Vienna, Austria; eva.ladenhauf@boku.ac.at (E.L.); dietmar.pum@boku.ac.at (D.P.)

**Keywords:** bovine serum albumin, quartz crystal microbalance, molecularly imprinted polymer, nanoparticles, associative effects

## Abstract

Herein we report novel approaches to the molecular imprinting of proteins utilizing templates sizing around 10 nm and some 100 nm. The first step comprised synthesizing nanoparticles of molecularly imprinted polymers (MIP) towards bovine serum albumin (BSA) and characterizing them according to size and binding capacity. In a second step, they were utilized as templates. Quartz crystal microbalances (QCM) coated with MIP thin films based on BSA MIP nanoparticles lead to a two-fold increase in sensor responses, compared with the case of directly using the protein as the template. This also established that individual BSA molecules exhibit different “epitopes” for molecular imprinting on their outer surfaces. In light of this knowledge, a possible MIP-based biomimetic assay format was tested by exposing QCM coated with BSA MIP thin films to mixtures of BSA and imprinted and non-imprinted polymer (NIP) nanoparticles. At high protein concentrations (1000 ppm) measurements revealed aggregation behavior, i.e., BSA binding MIP NP onto the MIP surface. This increased sensor responses by more than 30% during proof of concept measurements. At lower a BSA concentration (500 ppm), thin films and particles revealed competitive behavior.

## 1. Introduction

Molecular imprinting has established itself as one of the key techniques for generating biomimetic, selective receptor materials [1,2,3,4]. The resulting molecularly imprinted polymers (MIP) usually even exceed biological materials in terms of long-term stability [5] and low cost [3], and sometimes reach similar selectivity and sensitivity as their biological counterparts [2,6,7]. Whereas imprinting small molecules has become somewhat common practice [8], imprinting with macromolecules still poses challenges [9]. In the case of proteins, those are related to their larger size and limited stability and solubility in organic solvents [10]. On the other hand many popular monomers are insoluble or only partially soluble in water [11,12]. Generally speaking, surface imprinting has proven to be one of the most suitable approaches in the biomolecular imprinting of sensor receptor layers [13]. Hence, lower limits of detection of MIP-based protein sensors are normally higher than for biosensors [11,14]. Several attempts to tackle these obstacles have been reported, such as MIP nanofibers [15], nanoparticles [16], hydrogels [11,17], thin films of polymers [18] and epitope-imprinting techniques [19]. They rely on “straightforward” imprinting approaches. In this study, we aim at proving two novel imprinting concepts that show potential for developing actual MIP-based protein assay formats. Firstly, we rationally increased the surface roughness of protein MIP thin films. This was expected to lead to larger numbers of binding sites and hence more analyte-layer interactions on the surface. See, for example, [7,16,20,21,22]. For instance, in 2014, two papers demonstrated that nanoparticles can be used for molecular imprinting [23,24], which in turn, of course, led to an increase in surface. The present work takes this idea one step further by aiming at utilizing non-washed protein MIP nanoparticles (NPs) as templates instead of only the protein: we reasoned that this approach should lead to MIP thin films whose surface would be structured in two size ranges: some 100 nm through the nanoparticles, and 9–14 nm caused by BSA molecules immobilized on the surface of the MIP NPs. On the other hand we also aimed at proving an MIP-based detection concept that resembles current biosensors, namely associative binding. In that case, a protein molecule bound by MIP thin film is utilized to also bind MIP NPs to the film surface. Given the size of an average protein molecule, it is perfectly reasonable to assume that it can bind to two different MIP with two different faces. For assessing these approaches we used bovine serum albumin as a model compound and quartz crystal microbalance (QCM) measurements.

## 2. Materials and Methods

### 2.1. Chemicals and Reagents

All solvents and reagents were purchased in analytical grade. *N*-vinylpyrrolidone (VP), acrylic acid (AA), methacrylic acid (MAA), *N*,*N*′-(1,2-dihydroxyethylene) bisacrylamide (DHEBA), potassium peroxydisulfate (KPS), acetic acid, sodium dodecyl sulfate (SDS) and bovine serum albumin (BSA) were purchased from Merck (Darmstadt, Germany) and Sigma-Aldrich (Taufkirchen, Germany), respectively, and used as received.

### 2.2. QCM Fabrication

Quartz crystal microbalances (QCMs) were prepared by screen-printing the desired dual electrode structure with commercial brilliant gold paste (purchased from HERAEUS, Hanau, Germany) on an AT-cut quartz disc with a diameter of 13.8 mm and a thickness of 168 μm (purchased from Great Microtama Industries, Surabaya, Indonesia), following a previously developed procedure [25]. After printing, devices were heated to 400 °C for 4 h in order to remove organic residues (linseed oil) from the paste and to reveal the metallic gold electrodes. This procedure was repeated on the rear side of the quartz sheets. The electrodes facing the aqueous phase were electrically grounded. Their diameters were 5 mm, whereas the “backside” electrodes were 4 mm in diameter, to minimize conductivity effects. Such dual-electrode geometry consisting of a measuring electrode and a reference electrode, respectively, helps in eliminating non-specific effects caused by temperature, viscosity or non-selective adhesion.

### 2.3. Synthesis of MIP Thin Films

The MIP were based on materials that were previously developed in the group [10]: 35 mg of the cross-linker DHEBA, as well as 6 mg AA and 9 mg VP as functional monomers were dissolved in 800 µL distilled water. Then, 1 mg KPS was added as a radical initiator, and polymerization started under UV light until the gel point was approached. This pre-polymerized solution is suitable both for coating the respective transducer and for synthesizing nanoparticles.

**Figure 1:**
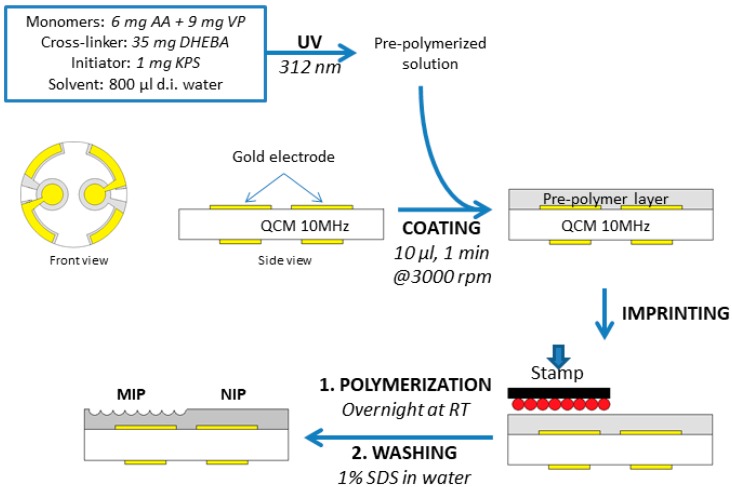
Schematic of BSA stamp imprinting. Abbreviations: AA, acrylic acid; DHEBA, *N*,*N*′-(1,2-dihydroxyethylene) bisacrylamide; KPS, potassium peroxydisulfate; QCM, quartz crystal microbalance; RT, room temperature; SDS, sodium dodecyl sulfate.

In parallel, template stamps were prepared by the sedimentation of the respective template on a microscope slide (5 mm × 5 mm), followed by drying at 4 °C for one hour. After that they were spinned at 3000 rpm for 2 minutes to remove excess solution. Table 1 summarizes all templates used for preparing MIP:

For generating MIP sensor layers (Figure 1), the pre-polymerized mixture was spin-coated onto a 10 MHz QCM at 3000 rpm. Immediately afterwards, the respective template stamp was pressed into the resulting layer. Both electrodes were coated with the same polymer, but only one of them was imprinted. Thus, the other one served as a reference based on the non-imprinted polymer (NIP), in order to compensate for physical and non-specific effects. Hence, one can make sure that imprinting is the reason for any difference in frequency shifts between the two channels. All polymer films were around 200 nm thick, which was determined both by atomic force microscopy (AFM) and by measuring frequency shifts. Any layers not inside the thickness range of 180–250 nm were discarded. The exact layer height is of no importance in surface imprinting, because signals are generated only on the polymer surface, not in the bulk. After coating, layers were hardened at room temperature (RT) overnight. Finally, templates were removed after polymerization by washing with a 1% (*w*/*w*) aqueous SDS solution and water.

### 2.4. Synthesis of MIP Nanoparticles

200 µL of pre-polymerized solution were mixed with 500 µL of aqueous BSA solution (5 mg/mL; to generate MIP NPs) or 500 µL of water, in the case of NIP NPs. Then, both solutions were treated under UV light (365 nm, 180 W) for 10 minutes followed by precipitation in 20 mL of acetonitrile with vigorously stirring overnight. This lead to precipitation of spherical particles sized between 70–250 nm as determined by AFM. NPs were then washed with solvent and centrifuged. These steps were repeated until the xanthoprotein reaction did not reveal any further protein in the particles.

### 2.5. QCM Measurement

The respective QCM was mounted in the cell, and exposed to a sample flow of 1 ml/min at 25°C (regulated by a thermostat). It acted as a frequency-determining element in an oscillator circuit operated by an input DC voltage source at 12 V and 60 mA. An Agilent 53131 A Universal Counter continuously monitored the oscillator circuit frequency, which was read out by a custom-made LabView routine into a computer. Sensors were sequentially washed with 1% SDS, 10% acid acetic, and water and then kept in water until a stable signal was reached. Subsequently, the sample solution was injected into the measuring cell. After reaching a constant frequency value, it was removed by flushing with 1% SDS and 10% acid acetic. Finally, the measuring chamber was flushed with water in order to reach baseline again.

## 3. Results and Discussion

### 3.1. Increasing Sensitivity of BSA MIP Thin Films

**Figure 2:**
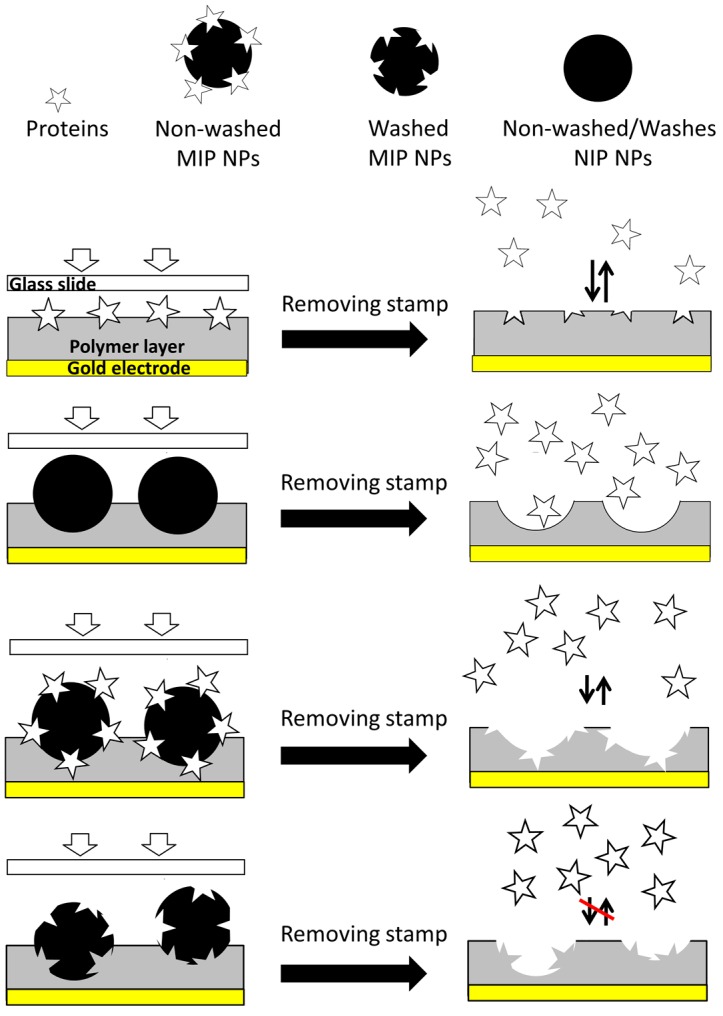
Different template strategies for BSA MIP. From top to bottom: MIP (BSA), MIP (NP–NIP), MIP (NP–MIP–BSA), and MIP (NP–MIP–wash).

Schirhagl et al. [26] demonstrated the possibility to transfer the recognition abilities of antibodies to polymer thin films by double imprinting: they first generated MIP nanoparticles toward insulin, then washed them and used them as templates in stamp imprinting. Since then, two more papers demonstrated that nanoparticles are useful templates [23,24]. Herein, we carry those approaches further by applying non-washed MIP NPs—MIP (MIP–NP–BSA) in Table 1—as templates, which we reasoned should lead to larger surface area and to a larger number of binding sites in the polymer. Figure 2 sketches the different imprinting approaches using BSA as the target analyte. Fully assessing the power of the approach requires four different templates, namely:

1. BSA as a reference value for the MIP thin film—MIP (BSA).
2. NIP nanoparticles to assess the contribution of increased film surface area—MIP (NP–NIP).
3. MIP nanoparticles directly after synthesis, i.e., still containing the template—MIP (NP–MIP–BSA).
4. Washed MIP nanoparticles—MIP (NP–MIP–wash).

As can be seen Figure 2, NIP NPs and washed MIP NPs were not suitable for the formation of cavities for selective BSA recognition.

**Figure 3:**
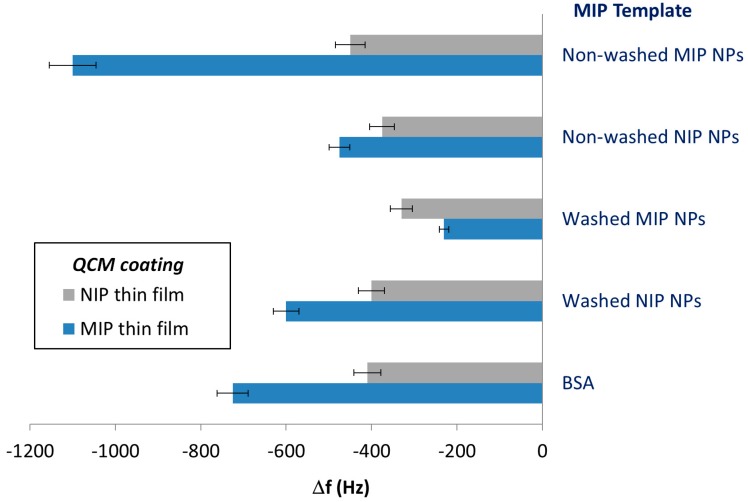
QCM sensor responses of MIP thin films resulting from different templates (specified in the right-hand column), respectively, and the NIP thin film towards 100 ppm BSA.

Figure 3 illustrates the outcome of this approach: the bottom two columns depict QCM sensor responses obtained with MIP (BSA) and NIP, respectively. Obviously, the MIP incorporated about twice as much protein as the NIP, thus clearly demonstrating imprinting effects. The MIP (NP–NIP) reveals a frequency shift towards BSA that is 1.5 times higher than that of NIP, namely −600 Hz compared to −400 Hz. This is the result of the increased surface area of the polymer film: Cavities left behind by NIP NPs on the surface are much larger than BSA and thus are not expected to selectively bind it. Even though MIP (NP–NIP) adsorbs more BSA than NIP, the sensor responses were still smaller than for the MIP (BSA) (−725 Hz vs. −600 Hz). Similarly, thin films imprinted with non-washed NIP NPs also lead to slightly increased sensor signals compared to NIP. The difference between washed and non-washed NIP NPs is that the former revealed slightly higher QCM sensor responses than the latter. This may be the consequence of the washing procedures, which often somewhat increase surface roughness.

MIP (NP–MIP–wash) thin films further decreased the sensor responses to BSA solutions (−230 Hz/−330 Hz for MIP and NIP, respectively). The reason is that washed MIP NPs contain cavities caused by BSA on their surfaces, so the imprinting procedure will form BSA “copies” on the surface of the thin MIP layer. Interestingly, this seems to decrease affinity toward BSA compared to the NIP surface. The effect is even more remarkable considering that BSA tends to form aggregates [27]. However, this is beyond the scope of this study.

The MIP (NP–MIP–BSA), finally, gave rise to higher imprinting effects than MIP (BSA), as can be seen in the uppermost column of Figure 3: Obviously, the sensor responses of MIP/NIP substantially increased to −1100/−450 Hz, compared to −725/−410 Hz (in the case of BSA as the template). The actual imprinting effect—i.e., the signal difference between MIP and NIP—was two times larger (315 Hz vs. 650 Hz). It is noteworthy that the signals of all NIP were rather similar. In general, this result properly supports the concept laid out in Figure 2.

**Figure 4:**
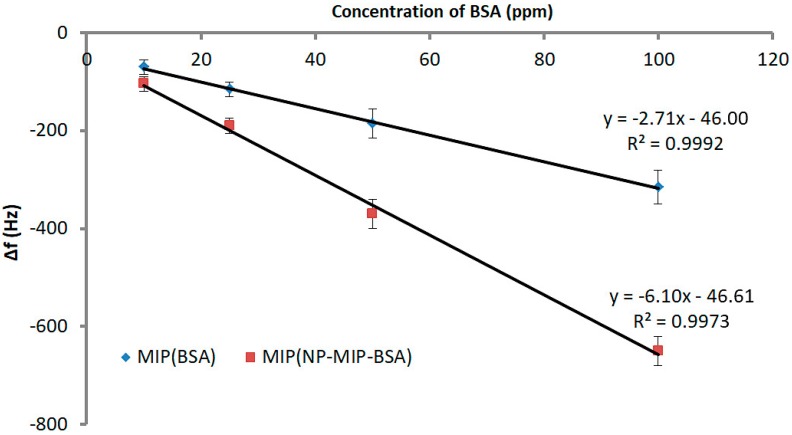
Sensor characteristics for MIP (BSA) and MIP (NP–MIP–BSA). Sensor responses represent the difference between MIP and NIP.

In a next step, we determined the sensor characteristics of both protein MIPs, namely MIP (BSA) and MIP (NP–MIP–BSA). Figure 4 summarizes the outcome in the concentration range from 10 ppm to 100 ppm. Evidently, replacing BSA as the template by the corresponding unwashed MIP NPs increased sensitivity—defined by the slope of linear regression—by more than a factor of two. Sensitivity was −6.10 Hz/ppm for layers templated with the non-washed MIP NPs, and −2.71 Hz/ppm for BSA MIP, and impressively demonstrates an MIP NPs strategy to increase the sensitivity in protein MIP. The corresponding limits of detection were LoD = 6 ppm for the BSA MIP and LoD = 4 ppm for the nanoparticle MIP.

Such effects can be traced back to the comparably large size of the proteins. Part of each BSA present on the surface of MIP NPs was exposed to solution. These “outer” surfaces can be utilized for imprinting. As the orientations of protein molecules on the MIP NP were statistically distributed, any part of BSA could be exposed on the outside. The cavity caused in the thin film by templating with these MIP NP is thus selective to another epitope of the same molecule. This is also in line with immunochemical reactions. A species/biomacromolecule usually contains several epitopes to which (different) antibodies can bind. In a future step it will be necessary to assess selectivity of the approach toward other proteins for this system, although recent BSA MIP have reported excellent selectivity [28,29,30]. Furthermore, previous experience with chemically related MIP matrices shows that they are much more rugged than biospecies. A MIP thin film selectively binding *E. coli* could be operated in a bioreactor for a month [31].

### 3.2. On the Way to MIP-Based Protein Assays

**Figure 5:**
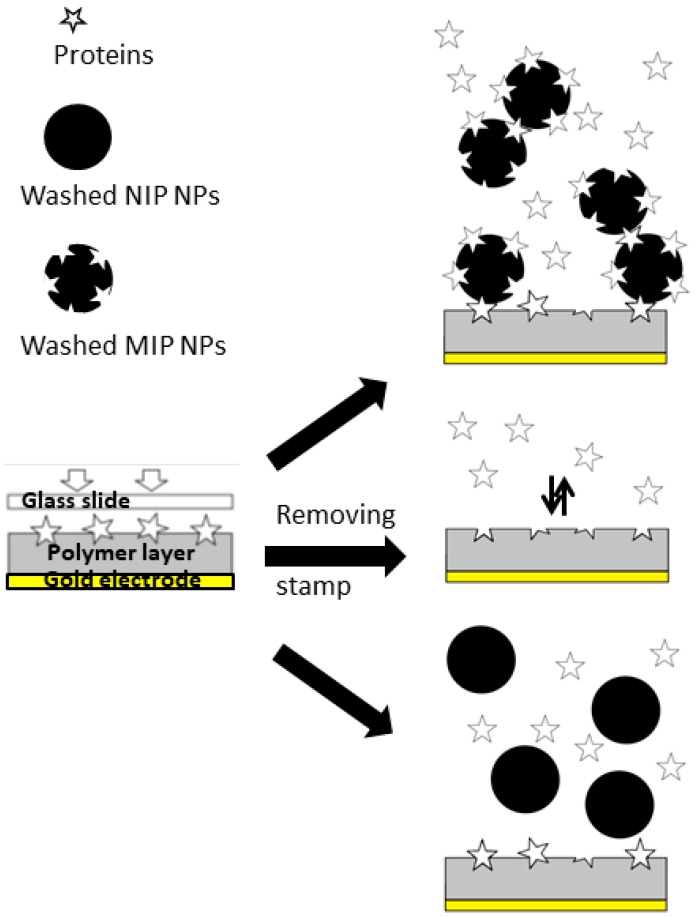
Experiment sketch for “cross-linking” MIP thin films and MIP NP by BSA molecules.

In order to further corroborate the assumption above, we set up several experiments as laid out in Figure 5:

For this purpose, we first exposed MIP (BSA) thin film to BSA, washed NIP NPs, and washed MIP NPs. In a second step we tested mixtures of BSA and washed NIP and MIP, respectively. During these proof-of-concept experiments, we utilized concentrated BSA solutions (500 ppm and 1000 ppm) to ensure the saturation of the binding sites of both sensor layers and nanoparticles. The concentration level was also chosen in order to ensure that sensor responses were significantly above noise level. The basic idea behind the experiment is the following: It is obviously possible to generate recognition sites for BSA in the MIP thin film by using unwashed MIP NPs. Hence it should also be possible to utilize BSA molecules for immobilizing the MIP MPs on the surface, i.e., using them as “linkers”.

**Figure 6:**
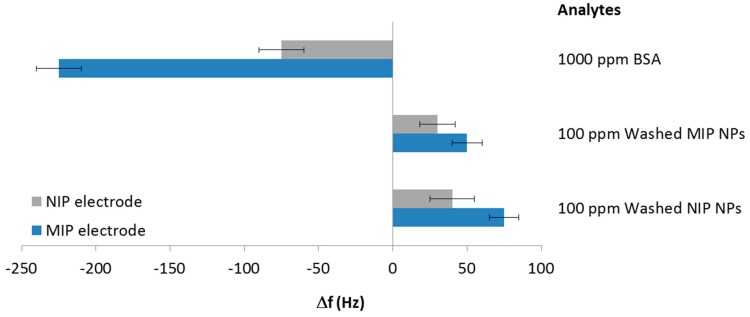
Response of MIP (BSA) sensors towards nanoparticles, and pure BSA.

The outcome of the first set of experiments is summarized in Figure 6: MIP (BSA) thin film led to almost three times higher frequency shifts toward 1000 ppm BSA solution than the corresponding NIP, thus clearly demonstrating the imprinting effect and the quality of this polymer as a BSA sensor layer. Exposing the same sensor layer to solutions containing washed MIP and NIP particles did not lead to selective binding. Both corresponding signal groups in Figure 6 reveal frequency shifts towards higher values, so-called Anti-Sauerbrey behavior. This can be explained by weak binding between nanoparticles and the comparably flat thin films, so that the nanoparticles retain some mobility on the sensor surface. Previous measurements with biospecies by Wangchareansak et al. [32] and Schirhagl et al. [33] have revealed similar effects on flat surfaces (mainly the respective NIP). As the diameters of possible recognition sites on the BSA MIP surface were much lower than those of the nanoparticles, the MIP can also be regarded as flat with respect to NP.

**Figure 7:**
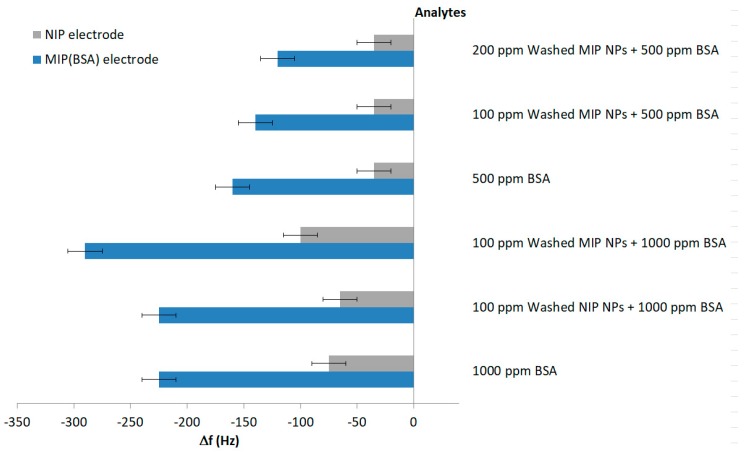
QCM Sensor responses of BSA MIP thin film and corresponding NIP towards different amounts of BSA and BSA/nanoparticle mixtures.

Figure 7 shows measurements of BSA sensors exposed to samples containing either only BSA, or mixtures of BSA with different nanoparticles. Adding NIP NP to BSA samples did not change the sensor responses at all. Both samples result in −225 Hz on the MIP channel. The effect on the different NIP signals (−75 Hz for BSA, −65 Hz for the mixture) were not statistically significant. QCM sensor responses were thus determined by BSA. Adding 100 ppm of washed NIP nanoparticles, however, revealed larger sensor responses, namely −290 Hz for the MIP. This strongly indicates that BSA indeed interacted with recognition sites on the MIP thin film and simultaneously with receptor sites on the nanoparticle surfaces. Additionally, in nanoparticle suspensions, BSA may become a cross-linker between nanoparticles, leading to larger aggregates, because its 3D structure may include two suitable binding sites—epitopes—on each “face”. Thus, the MIP thin films on QCM bound to both individual BSA molecules and agglomerates. The effect was not very large, but statistically significant: particles without BSA led to non-Sauerbrey effects on the QCM (see Figure 6). NIP layers also give rise to similar phenomena, however, only slightly so (−100 Hz compared to −75 Hz of pure BSA). Aggregates of BSA cross-linking two nanoparticles may have been the reason for this.

Interestingly, sensor responses looked different when decreasing BSA concentration to, for example, 500 ppm. Figure 7 shows that every extra 100 ppm of washed MIP nanoparticles added to a solution containing 500 ppm BSA reduced the MIP signal by 20 Hz, whereas NIP signals did not change at all. Obviously, part of BSA in solution was bound to nanoparticles instead of interacting with the sensor layer. However, it is not sufficient to aggregate these nanoparticles in order to increase the signal, like in the case of 1000 ppm BSA solution. That also is why the response of NIP was not altered by these changes of nanoparticles concentration. In practice, the mixture of washed MIP NPs and 1000 ppm BSA precipitated faster than the one containing 500 ppm BSA (2 h compared to 6 h). Obviously, the amount of agglomerates in the former solution was larger than in the latter.

## 4. Conclusions

The present study revealed that combining protein MIP thin films nanoparticles increases sensitivity in two different ways:

1. Stamps comprising unwashed BSA MIP NPs can be utilized as templates instead of BSA to generate surface imprints on polymer thin films. The resulting layers—MIP (MIP–NP–BSA)—give rise to two times higher QCM sensor responses than corresponding MIP (BSA).
2. At higher BSA concentrations, the protein can be utilized to bind MIP NPs to MIP thin film surfaces. In principle, this makes MIP-based, biomimetic assays possible where NPs are utilized for amplifying the sensor signals from binding.

Both phenomena rely on the comparably large size of protein molecules. When synthesizing BSA MIP nanoparticles, the template generates imprints on the respective nanoparticle surface. To exhibit them, BSA needs to be washed off. Until then, BSA molecules remain bound to the surface of each BSA MIP NP. These molecules are statistically oriented on the surface. Therefore any MIP particle contains a number of different BSA “epitopes” on its surface. Which are useful for imprinting. These “epitopes” can, in turn, also bind to a recognition site on a BSA MIP surface (be it thin film, or nanoparticle) thus effectively cross-linking the two polymers. Such collaborative effects are observed at comparably large protein concentrations (1000 ppm). Decreasing it to 500 ppm leads to competitive effects between MIP NPs in solution and the MIP surface, thus effectively reducing the QCM signals of the MIP thin film. This, in principle, opens the way for MIP-based agglutination assays. To the best of our knowledge, both concepts have been shown for the first time herein.

For actual applicability in analysis, it will be necessary to increase the sensitivity of the materials and to synthesize MIP NPs containing, ideally, only one binding site per particle in strategies similar to those recently suggested by the groups of Piletsky [34] and Haupt [35].

## Figures and Tables

**Table 1:** Summary of templates used. Abbreviations: NIP, non-imprinted polymer; MIP, molecularly imprinted polymers; BSA, bovine serum albumin; NP, nanoparticle.

Recognition Element	Template
NIP	None
MIP (BSA)	10 µL BSA solution (50 mg/mL)
MIP (NP-NIP)	10 µL of a solution containing 10 mg/L NIP nanoparticles
MIP (NP-MIP-BSA)	10 µL of a solution containing 10 mg/L MIP (BSA) nanoparticles after synthesis, i.e., still containing BSA molecules on their surfaces
MIP (NP-MIP-wash)	10 µL of a solution containing 10 mg/L MIP (BSA) nanoparticles after washing, i.e., after removing BSA molecules
